# Innate and Adaptive Immunity in Giant Cell Arteritis

**DOI:** 10.3389/fimmu.2020.621098

**Published:** 2021-02-25

**Authors:** Mitsuhiro Akiyama, Shozo Ohtsuki, Gerald J. Berry, David H. Liang, Jörg J. Goronzy, Cornelia M. Weyand

**Affiliations:** ^1^ Department of Medicine, Stanford University School of Medicine, Stanford, CA, United States; ^2^ Department of Pathology, Stanford University School of Medicine, Stanford, CA, United States

**Keywords:** T cell, macrophage, vasculitis, NOTCH, endothelial cell, PD-L1, CD8 Treg, exosome

## Abstract

Autoimmune diseases can afflict every organ system, including blood vessels that are critically important for host survival. The most frequent autoimmune vasculitis is giant cell arteritis (GCA), which causes aggressive wall inflammation in medium and large arteries and results in vaso-occlusive wall remodeling. GCA shares with other autoimmune diseases that it occurs in genetically predisposed individuals, that females are at higher risk, and that environmental triggers are suspected to beget the loss of immunological tolerance. GCA has features that distinguish it from other autoimmune diseases and predict the need for tailored diagnostic and therapeutic approaches. At the core of GCA pathology are CD4^+^ T cells that gain access to the protected tissue niche of the vessel wall, differentiate into cytokine producers, attain tissue residency, and enforce macrophages differentiation into tissue-destructive effector cells. Several signaling pathways have been implicated in initiating and sustaining pathogenic CD4^+^ T cell function, including the NOTCH1-Jagged1 pathway, the CD28 co-stimulatory pathway, the PD-1/PD-L1 co-inhibitory pathway, and the JAK/STAT signaling pathway. Inadequacy of mechanisms that normally dampen immune responses, such as defective expression of the PD-L1 ligand and malfunction of immunosuppressive CD8^+^ T regulatory cells are a common theme in GCA immunopathology. Recent studies are providing a string of novel mechanisms that will permit more precise pathogenic modeling and therapeutic targeting in GCA and will fundamentally inform how abnormal immune responses in blood vessels lead to disease.

## Introduction

Giant cell arteritis (GCA), also known as “temporal arteritis,” is an autoimmune disease that exclusively affects the elderly host (1). The disease preferentially involves the thoracic aorta and its major branch vessels, including the temporal artery and vessels supplying the optic nerve and the retina. Accordingly, the clinical manifestations of GCA include life-threatening complications, such as aortic dissection, aortic aneurysm, and blindness due to ischemia of the optic nerve. Globally, the highest incidence rates of GCA occur in Northern Europe, including Iceland, Norway, Sweden, and Denmark. High disease risk in Northern European populations has supported the concept that both genetic and environmental factors shape disease susceptibility. Genome-wide association studies have confirmed earlier data that polymorphisms in the major histocompatibility complex (MHC), specifically the *Human leukocyte antigen (HLA)-DR* region confers the highest risk (2, 3). Amongst non-HLA regions, *PLG* and *P4HA2* appear to play a role as risk determinants (**Table 1**) (4). PLG (plasminogen) and P4HA2 (Prolyl 4-hydroxylase subunit alpha-2) are involved in vascular remodeling and neoangiogenesis, suggesting relevance of these processes in GCA pathogenesis. Of interest, a distinct set of genetic polymorphisms have been implicated in Takayasu arteritis (TAK) (5, 6), an autoimmune large vessel vasculitis that shares many similarities with GCA but preferentially affects young Asian women. In TAK, *HLA-B* has been shown to have the strongest disease association (**Table 1**). Like in GCA, patients with TAK have enrichment of genetic polymorphism in non-HLA regions; include such functionally related to activation of cytotoxic lymphocytes, e.g. natural killer cells and CD8^+^ T cells. Differences in disease risk genes in GCA and TAK indicate that different pathomechanisms may contribute to autoimmune and auto-inflammatory diseases of the large arteries (7–9).

**Table 1:** Gene regions associated with large vessel vasculitis.

Reported year	Population	The number of participants	Gene region
2017	European ancestries	2134 GCA patients 9125 controls	*HLA-DRA/HLA-DRB1, PLG*, *P4HA2*
2018	Japan	415 TAK patients 2170 controls	*HLA-B*, *FCGR3A*, *IL12B*, *DUSP22*, *PTK2B*, *KLHL33*, *LILRA3*, *chr21q22*
2015	Turkey and North America	693 TAK patients 1536 controls	*HLA-B/MICA*, *IL6*, *RPS9/LILRB3*, *chr21q22*

**Figure 1:**
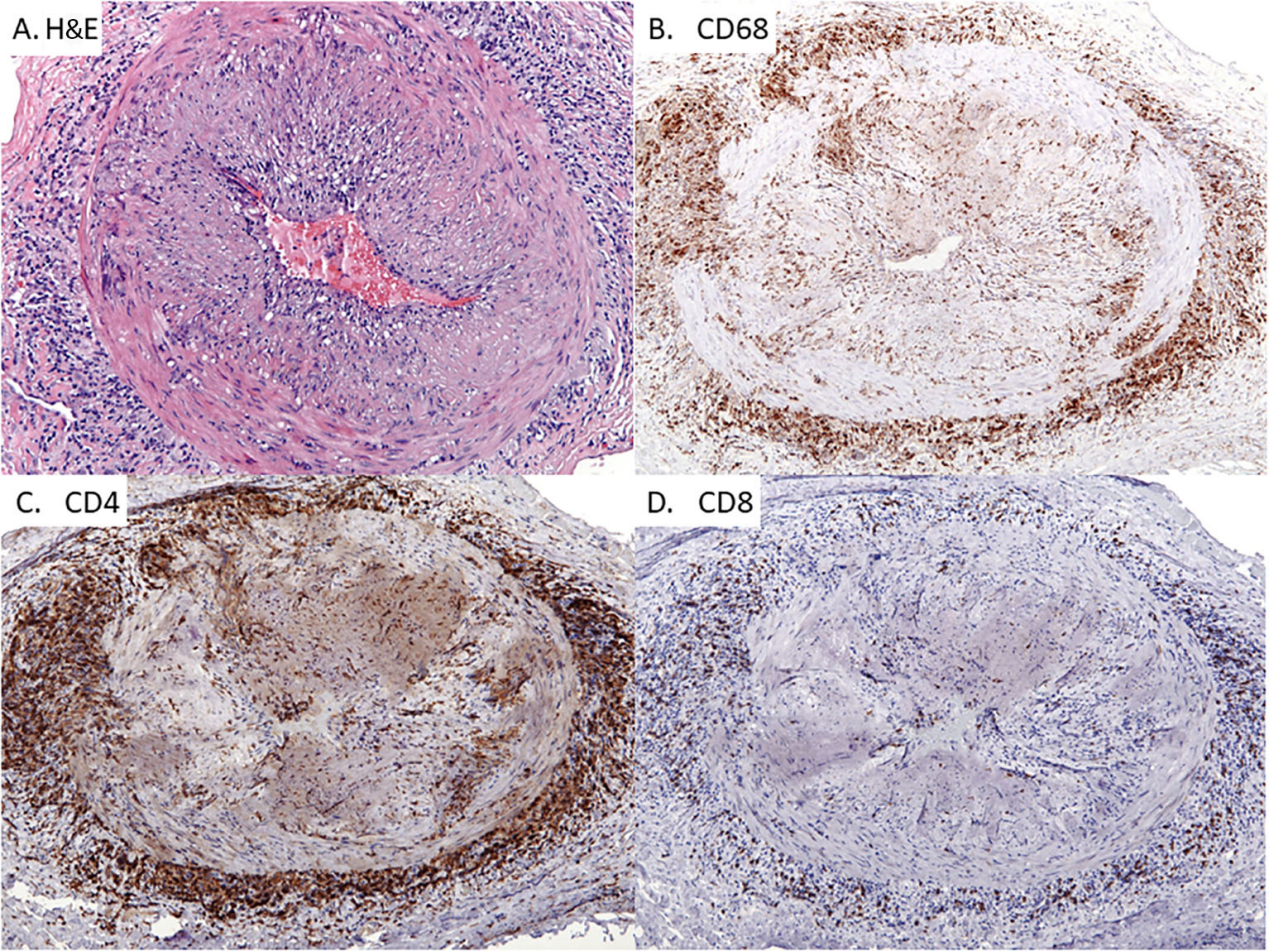
Giant Cell Arteritis in the Temporal Artery. Classic histopathologic and immunophenotypic findings of temporal arteritis in a 78-year old woman presenting with headaches. Panel **(A)** Transmural arterial inflammation with marked luminal narrowing caused by intimal proliferation which creates a slit-like lumen. Multinucleated giant cells are concentrated in the medial layer (H&E x125) Panel **(B)** CD68-positive histiocytes accumulating within the medial and adventitial layers of the artery with only scattered histiocytes in the intimal layer (x100). Panel **(C)**: CD4^+^ T cells with a similar pattern of distribution as the CD68^+^ macrophages (x100). Panel **(D)** Infrequent CD8^+^ T cells within the T cell infiltrates (x100).

The vasculitic lesions of GCA are composed of tissue-infiltrating and tissue-resident innate and adaptive immune cells; mostly, CD4^+^ T cells, dendritic cells, macrophages, histiocytes, and multinucleated giant cells (10, 11) (**Figure 1**). Recent advances in understanding the pathogenesis of GCA have provided important insights into disease-inducing and -sustaining mechanisms. Key pathogenic elements include a vascular and an extravascular disease component, with site-specific immune processes relevant for disease inside and outside of the vascular wall. Also, it is now appreciated that the vessel wall has unique structural barrier features that make it an immuno-privileged tissue site, protecting it from unwanted immune responses. Breakdown of this immune privilege require aggressive immune responses that first must overcome the natural protection inherent to life-sustaining arteries. Studies of persistent vasculitis in GCA patients have stressed the autonomy of tissue-residing inflammatory infiltrates, building significant challenges for the elimination of vascular wall inflammation. Another hurdle in treating GCA relates to the functional heterogeneity of vasculitic effector cells, which lends stability to the inflammatory lesions and renders them resistant to targeted immunosuppression. The granulomatous nature of the vessel wall lesion has nurtured discussions that infectious microorganisms may serve as the vasculitogenic antigen, but reproducible data implicating a viral or bacterial antigen are missing (9). Other environmental triggers, such as air pollutants etc are insufficiently explored. The extravascular component of GCA is poorly understood. Clinically and diagnostically, it is characterized by intense acute phase responses, resulting in elevated Erythrocyte Sedimentation Rate (ESR) and C-reactive protein (CRP). Patients also complain about constitutional symptoms and proximal myalgias that are promptly responsive to glucocorticoids and the recently approved anti-interleukin (IL)-6 receptor antibody tocilizumab (12). However, fluctuations in acute phase reactants, as captured by measurement of ESR and CRP, can occur despite persistence of vessel wall inflammation (13). The lack of reliable disease biomarkers capturing vessel wall inflammation is problematic in managing GCA.

The present review will focus on recent advances in understanding the dysfunctional innate and adaptive immune responses that cause autoimmune vasculitis, with a focus on how the arterial wall immuno-privilege is broken, how vascular inflammation is sustained and how abnormal immunity maintains vascular remodeling. Progress in understanding pathogenic cascades will inevitably broaden the therapeutic armamentarium that is so urgently needed to improve management of vasculitis.

## Innate Immunity in GCA

### Monocytes and Macrophages as Disease Drivers in GCA

The three-layered walls of the muscular and elastic arteries are free of inflammatory cells and protected by immune privilege. Inaccessible tissue sites, e.g. in the testis and the eye, prioritize the integrity of life-sustaining organs over localized immunity. Maintenance of such privileged sites involve a combination of mechanisms, including physical barriers, lack of antigen-presenting cells and counter regulatory processes dampening immune stimulation. In the case of GCA, the immune privilege is lost and both, innate as well as adaptive immune cells enter the privileged site (**Figure 1**). In the three-layered arteries, composed of the intimal layer, the medial smooth muscle cell layer and the supportive adventitial layer, access to the vessel wall occurs through the adventitial vasa vasorum network.

**Figure 2:**
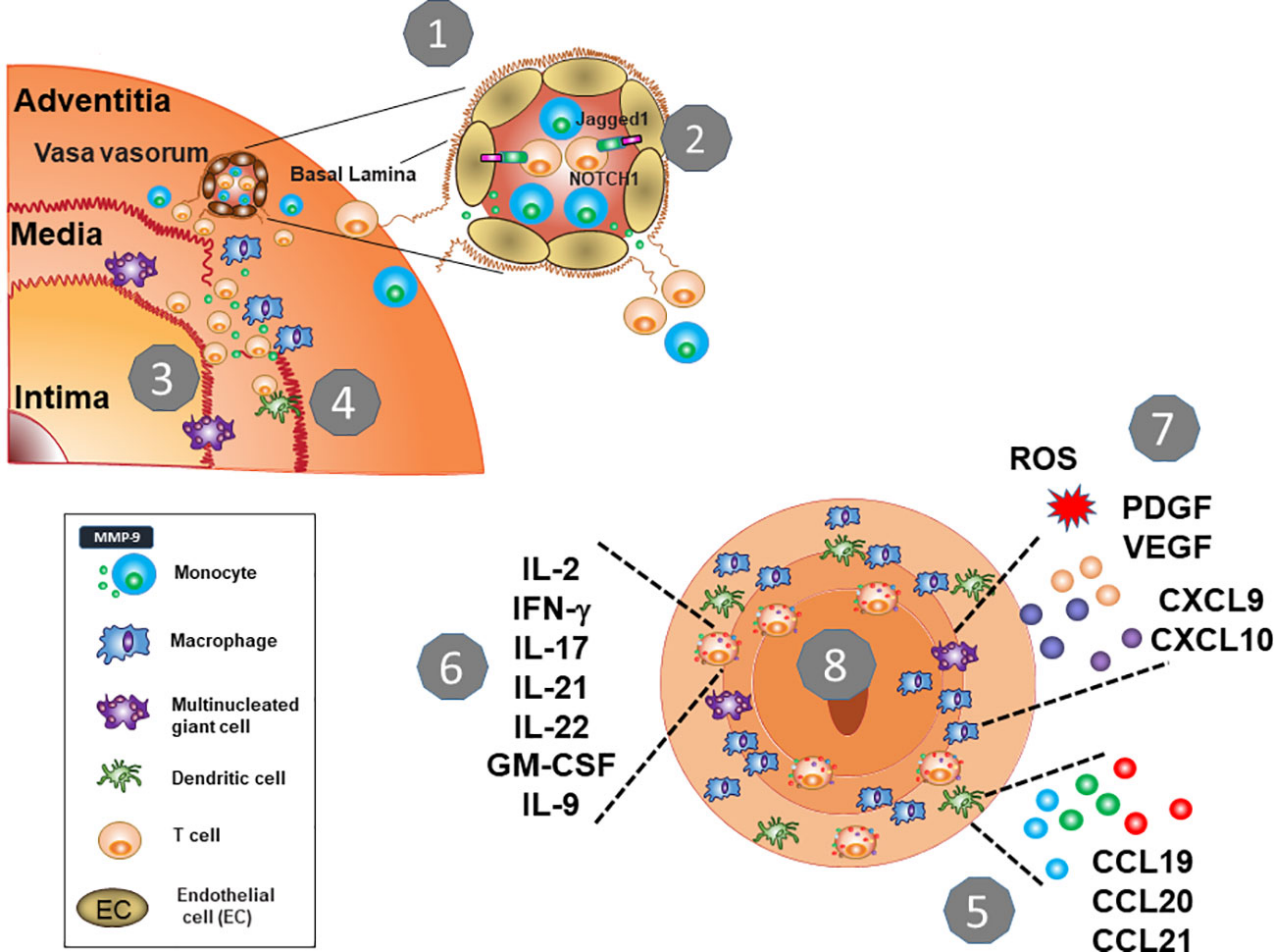
Key Pathogenic Steps in Giant Cell Arteritis. **1.** The protective shield of the artery wall breaks when immune cells leave the vasa vasorum and invade into the tissue. An essential checkpoint is the digestion of the vascular basal lamina, facilitated by MMP-9-producing monocytes. **2.** Vasculitic T cells follow, and macrophages (histocytes) and T cells form granulomatous infiltrates in the adventitia and media. **3.** MMP-9-producing macrophages destructs the elastic laminae and eventually, tissue-damaging multinucleated giant cells emerge. **4.** T cells encounter DC that lack the immunoinhibitory ligand PD-L1 and enter unopposed and persistent activation. **5.** Wall-residing DC provide chemokines and cytokines to enhance immune cell recruitment. **6.** Chronically stimulated T cells differentiate into multifunctional effector cells providing an array of effector cytokines. They also acquire tissue residence and replenish the lesion from within. **7.** Macrophages are functionally heterogenous, but their functional commitment is directly related to their positioning in the vessel wall. Macrophage products include chemokines and cytokines, tissue-damaging mediators (ROS, MMP-9) as well as growth factors (VEGF, PDGF). **8.** Continuously stimulated T cells and macrophages elicit a maladaptive response-to-injury presenting as vessel wall remodeling, with wall vascularization and intimal hyperplasia.

Recent work has described a molecular defect in circulating blood monocytes from GCA patients, which endows these cells with tissue invasive capabilities. Specifically, GCA monocytes spontaneously produce high amounts of the metalloproteinase MMP-9 and digest the basement membrane to overcome the barrier between vasa vasora capillaries and extracellular tissue. By blocking the proteinase activity of MMP-9 with a monoclonal antibody, Watanabe et al (14). implicated GCA monocytes in the breakdown of the basement membrane and in paving the way for both innate and adaptive immune cells into the wall (**Figure 2**). Remarkably, T cells failed to invade the tissue site unless they were accompanied by MMP-9-producing monocytes. Besides MMP-9, GCA monocytes produced high amounts of MMP-2 and -7 transcripts, while MMP-1, -3, -8, -10, or -12 transcripts were indistinguishable in GCA and control monocytes. Metalloproteinases are critically important in several physiologic and pathologic processes (15, 16) and it likely that inappropriate MMP-9 production is not an exclusive problem in GCA. However, the upregulation of MMP-9 already in monocytes clearly distinguishes GCA patients from healthy individuals.

**Figure 3:**
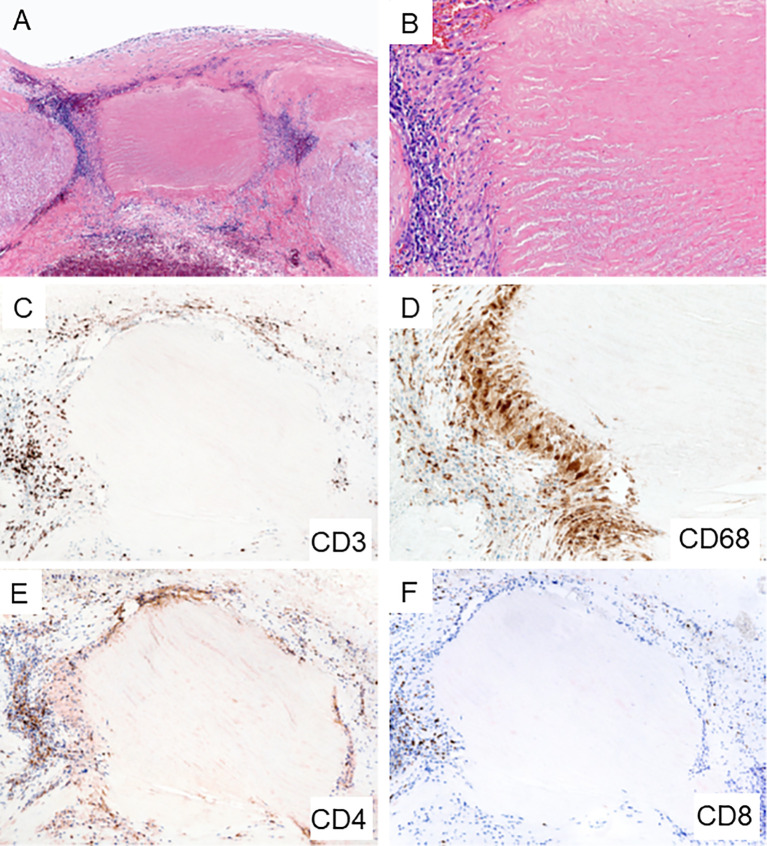
Giant Cell Arteritis in the Aorta. Biopsy sample from surgically removed aortic wall of a patient undergoing emergency aortic repair. **(A, B)** Hematoxylin and eosin staining showing typical granulomatous inflammation with rings of predominantly lymphocytes and macrophages around necrotic medial tissue (A x60; B x200). **(C)** CD3^+^ T cells form a collarette of inflammation enclosing the necrotic aortic wall (x100). **(D)** CD68^+^ histiocytes palisade at the edge of the damaged tissue (x200). **(E)** CD4^+^ T cells are the dominant T cell subset within the granulomatous infiltrates (x100). **(F)** Infrequent CD8^+^ T cells in the aortic wall (x100).

Once circulating monocytes have differentiated into macrophages, the propensity to produce excess MMP-9 continuously is a distinguishing feature of macrophages that reach the adventitial and medial layer of the artery (14). Also, multinucleated giant cells (MNG), the hallmark cell of GCA lesions, have a particularly strong signal for MMP-9, indicating that the metalloproteinase critically affects events that lead to the formation and the destructive potential of the granulomatous lesions (**Figure 2**). A typical feature of GCA is the thinning of the medial layer, presumably by injuring vascular smooth muscle cells and the fragmentation of the elastic laminae that separate the media from the intima. Here, local delivery of MMP-9 by tissue-invasive monocytes/macrophages emerges as a pinnacle event. The media of a healthy artery is essentially impenetrable, a barrier that must be overcome by pathogenic macrophages endowed with MMP-9-dependent elastolytic and gelatinolytic activities (17, 18). Thus, abnormal programming of monocytes represents an upstream disease element, facilitating initial entrance and maneuvering of inflammatory cells in the arterial wall. This pathomechanism may be particularly important in the aorta of GCA patients, prone to dissection and aneurysm formation (**Figure 3**).

How aberrant MMP-9 production in GCA monocytes and macrophages is induced has remained unresolved, but this is a defect that is a prerequisite of disease and is present early in the disease process. MMP-9 continues to participate in the granulomatous inflammation in established and in late disease (**Figure 2**). The aberrant production of MMP-9 in monocytes and macrophages of GCA patients seems to be combined with upregulation of MMP-2, suggesting a coordinated pathomechanism that affects families of enzymes (14, 19).

Macrophage populations that settle in the granulomatous lesions are highly heterogenous. Early studies have given rise to the concept that geographical mapping of the macrophages and functional commitment are linked. Macrophages in the media and at the media-intima border are most disease relevant. The fragmentation of the lamina elastica interna, the formal landmark separating the vascular smooth muscle cell (VSMC)-rich media from the intima, remains a hallmark of disease. The tissue-destructive potential of medial macrophages rests on the production of MMP-9, but also on the release of reactive oxygen species (ROS) (20). The tissue-damaging features of medial macrophages are counterbalanced by their ability to provide growth factors and angiogenic cytokines (**Figure 2**). Platelet-derived growth factor (PDGF)-producing macrophages and multinucleated giant cells sitting at the fragmented elastic lamina are critically important in driving the wall remodeling process, including the growth of neointima (21). Several cell types, including myofibroblasts, dedifferentiated VSMCs, and mesenchymal stem cells fuel the formation of the neo-tissue that occupies the artery’s lumen and blocks blood flow (22). PDGF has been implicated in facilitating proliferation and directed migration of precursor cells. This maladaptive wound healing process is only possible with sufficient neoangiogenic activity. While intramural vessels are usually restricted to the adventitia, inflamed temporal arteries contain networks of newly formed capillaries, penetrating the media as well as the intima (21). Here, macrophage-derived vascular endothelial growth factor (VEGF) is instrumental in providing a potent growth factor for endothelial cells (23). Interestingly, multinucleated giant cells possess the ability to synthesize VEGF. VEGF is elevated markedly in the circulating blood of GCA patients (24–26), where this angiogenesis factor functions as a regulator of endothelial cells and promotes endothelial cell-T cell interaction (24). However, the precise cellular source of the circulating VEGF in GCA patients has not been determined. A close correlation between tissue site and macrophage functional commitment in the vasculitic lesions has recently been confirmed by Jiemy et al, who showed that MMP-9^+^ macrophages were placed at areas of tissue destruction, while FRβ+ macrophages were positioned in the hyperplastic intima (27).

GCA is not the only vasculitis in which macrophages are key pathogenic effector cells. Rather, accumulation of highly activated macrophages in the disease lesions is a feature of other granulomatous diseases, including the small vessel vasculitis granulomatosis with polyangiitis (GPA). Specifically, overrepresentation of MMP-9 producing macrophages appears to be common between GCA and GPA (28). GPA is an autoimmune vasculitis of small blood vessels, typically associated with tissue destruction due to granuloma formation. Neutrophils forming neutrophil extracellular traps (NETs) are believed to function as an inflammatory nidus. In a subset of GPA patients the disease predominantly manifests in the head and neck (H&N), presenting with bony erosions of the orbital and sinus walls, septal perforations, saddle-nose deformities, middle ear damage and epiglottitis, all related to uncontrolled destruction of bone, cartilage, and connective tissues. In mechanistic studies, NETs released from H&N GPA neutrophils functioned as powerful stimulators of macrophages, inducing MMP-9 production (28). Such MMP-9 high-producing macrophages possess tissue-destructive capabilities (28), and MMP-9-producing macrophages and multinucleated giant cells dominate the granulomatous tissue infiltrates in naso-sinal biopsies from H&N GPA patients (28). These data implicate degradation of collagen IV in basement membranes and digestion of extracellular matrix in the pathologic events leading to GPA.

Given the similarities in GCA and GPA, dysregulation of MMP-9 production may be a fundamental pathomechanism, shared amongst vasculitides and shared by diseases with granulomatous inflammation. So far, none of the genetic polymorphisms predisposing to either GCA or GPA have been connected to the functional domain of metalloproteinases.

Macrophages from GCA patients have been functionally compared to those of another vasculopathy, namely coronary artery disease (CAD). CAD is now accepted as an inflammatory blood vessel condition that progresses over decades and causes the formation of atherosclerotic plaques in the subendothelial space of susceptible arteries (29). In the atherosclerotic plaque, highly activated macrophages take up deposited lipoproteins and modified lipids to transform into the pathognomonic foam cells. Giant cell formation occurs in just a subset of patients with atherosclerotic lesions. Thus, in GCA and CAD macrophages perform fundamentally different functions. Comparison of monocytes and monocyte-derived macrophages from GCA and CAD patients has demonstrated that these myeloid cells have distinct molecular signatures. CAD macrophages are prone to produce high amounts of inflammatory cytokines, such as IL-1β and IL-6, even more so than GCA macrophages (30). Another distinguishing features between the two diseases is the expression of the co-inhibitory ligand PD-L1, which is distinctly low in GCA, but high in CAD (30). Notably, macrophages from both patient populations abundantly produced chemokines (CXCL9, CXCL10), supporting a role in cell recruitment and assembly of the vessel wall lesions (**Figure 1B**). Metabolic conditioning was identified as the underlying mechanism. While CAD macrophages were programmed to uptake and utilize glucose, this was not the case for GCA macrophages. Addiction to glucose is one of the driving forces in CAD macrophages, dictating the dynamics of the glycolytic pathway, the setting of mitochondrial activity, the production of reactive oxygen species and ultimately, the secretion of IL-6 (31). The low glycolytic activity in GCA monocytes may be part of a broader metabolic program, as fasting blood glucose, cholesterol and triglyceride levels have been described to be negatively associated with the development of giant cell arteritis (32).

In summary, “trained immunity” in GCA leads to monocyte instruction, changing their metabolic circuitry and their functional differentiation. The concept of “trained immunity” is well understood in non-vasculitic cardiovascular disease (33–36) and relates to the concept that monocytes, macrophages, dendritic cells, and NK cells can be imprinted by encountering inflammatory stimuli, undergoing a priming process that changes their response to subsequent challenges. It is now recognized that the “training” is imprinted into the epigenome. In GCA monocytes, a lead abnormality is the high expression of MMP-9, a protease that takes center stage when inflammatory cells leave the blood stream and enter the “forbidden territory” of the vessel wall. Also affected is the expression of co-inhibitory ligands and the commitment to cytokine production. The training of monocytes has profound consequences for their later life as macrophages. They continue to produce MMP-9, now enabling them to destroy the tissue microenvironment. Functional analysis of lesional macrophages has emphasized their tissue repair capabilities, including the production of growth and angiogenesis factor, all promoting the maladaptive remodeling process in the GCA-affected artery (**Figure 2**).

### Vascular Dendritic Cells (DC) as Presenters of Vasculitogenic Antigens

**Figure 4:**
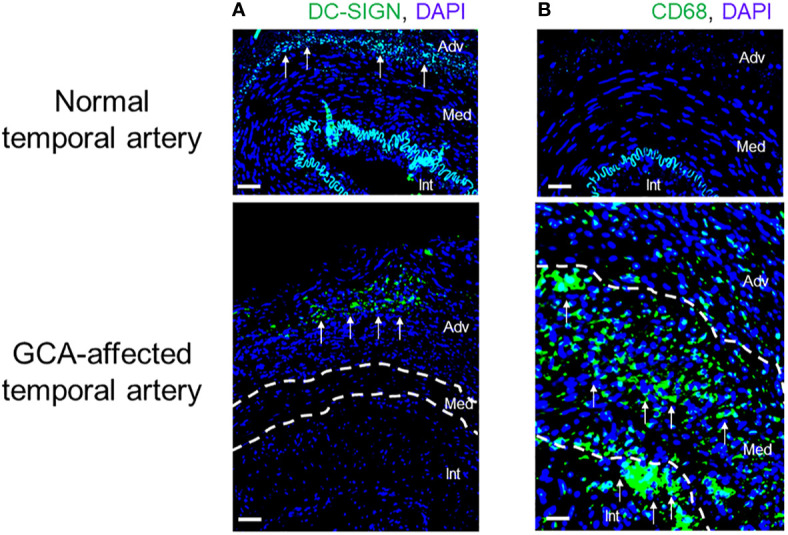
Innate Immune Cells in Giant Cell Arteritis. Tissue sections from temporal artery biopsies were stained for the dendritic cell (DC) marker DC-SIGN **(A)** and the macrophage marker CD68 **(B)** and visualized by immunofluorescence imaging. Nuclei marked by DAPI. In the healthy artery, the autofluorescent lamina elastica interna separates the media and intima. DC-SIGN^+^ dendritic cells are positioned at the adventitial-medial border. In the vasculitis-affected artery, DC-SIGN^+^ dendritic cells expand in the adventitia. CD68^+^ macrophages are essentially undetectable in the healthy artery but occupy all wall layers of the GCA artery. Int, intima; Med, media; Adv, adventitia. Scale Bar; 50 μm.

**Figure 5:**
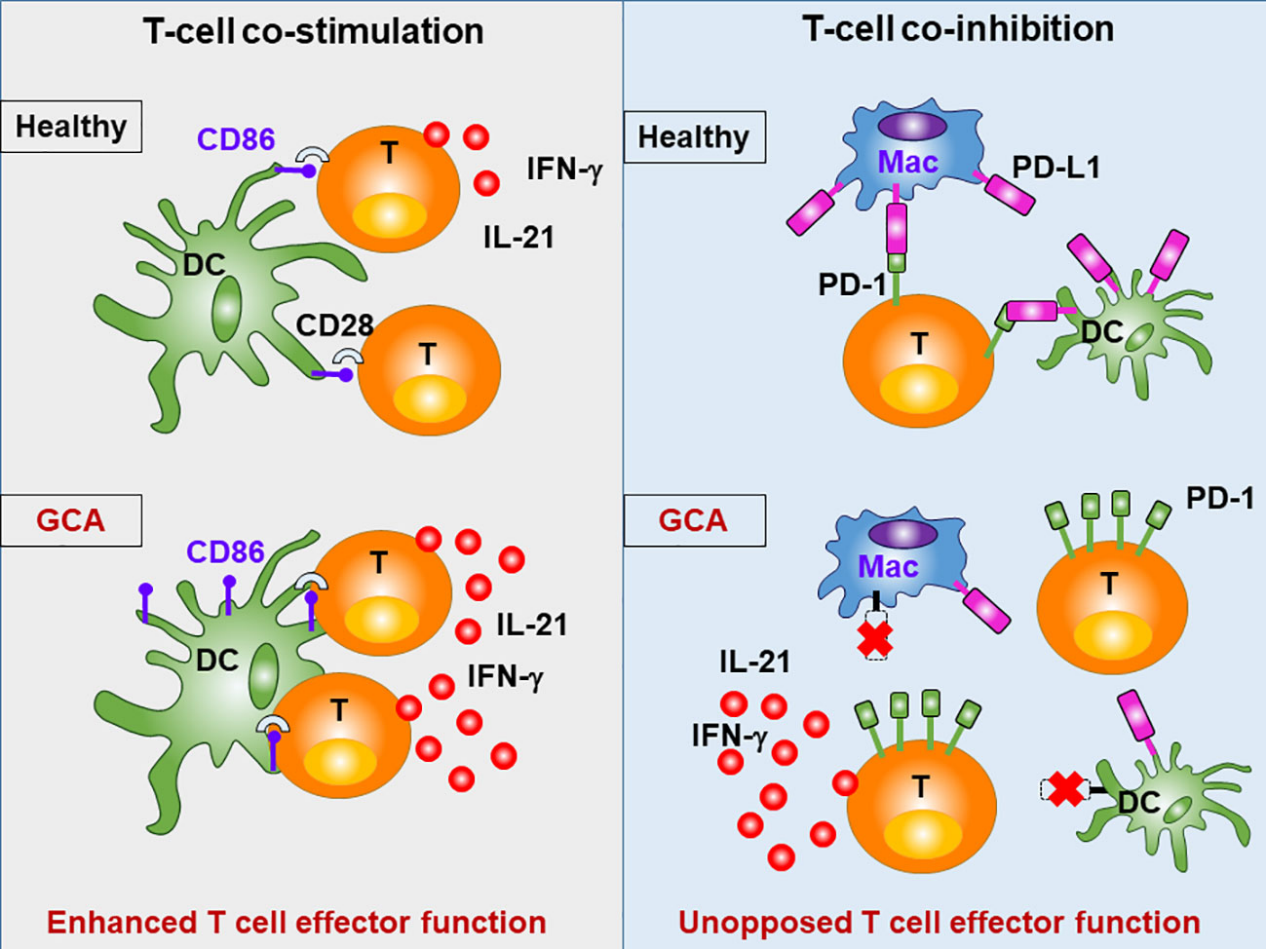
Abnormal T cell Activation in GCA. The intensity and duration of adaptive immunity depends on the availability of specific antigen, but also on a mixture of positive (co-stimulatory) and negative (co-inhibitory) signals, that modulate the T cell receptor activation cascade. Patients with GCA have abnormalities in the CD28 co-stimulatory pathway and in the co-inhibitory PD-1/PD-L1 pathway, resulting in sustained and unopposed activation of pathogenic T cells. Under physiologic conditions, CD28 on T cells recognizes CD80/86 on antigen-presenting cells (e.g. dendritic cells; DC), prolonging and intensifying T cell activation. Signaling through this pathway is intensified in GCA. Under physiologic conditions, PD-1 on T cells recognizes PD-L1 on antigen presenting cells (e.g. macrophages; Mac), resulting in dampening of T cell activation. In GCA, PD-L1 is expressed at very low levels, disrupting this negative signal, and boosting T cell effector functions.

DCs are part of the innate immune system and are indispensable for the induction of adaptive immune responses. Specifically, DCs are needed to present antigen for T cell priming and are thought to be the principal initiators of T cell immunity (37, 38). Besides their role in presenting exogenous antigens, such as microbial antigens and allergens, DCs are also instrumental in the handling of self-antigens and thus determine the fate of auto-reactive T cells. In addition, activated DCs are an important source of cytokines and chemokines, orchestrating the assembly of inflammatory infiltrates. Finally, they finetune T cell activation by providing both co-stimulatory and co-inhibitory signals for T cells (37, 38). Critically involved in activating naïve T cells, DCs function in secondary lymphoid organs, such as lymph nodes and the bone marrow. In the case of medium and large arteries, they possess their own tissue residing DCs, so-called vasDCs (39, 40) (**Figure 4**). Such vasDCs are believed to have two disease relevant functions in GCA; (a) guarding the vessel wall immune privilege, possibly by providing tolerogenic signals, and (b) presenting vasculitogenic antigens in the vessel wall infiltrates (39, 40). Healthy temporal arteries possess vasDCs positioned at the adventitia-media junction (**Figure 4**). In the inflamed artery, DCs may move into other tissue niches to join macrophages in presenting antigen to T cells that are distributed throughout the vessel wall. vasDCs placed in the granulomatous infiltrates produce chemokines, such as CCL19, 20, and 21, and strongly express the co-stimulatory molecule CD86 (41–43) (**Figure 5**). The disease relevance of the CD28-CD86 co-stimulatory pathway was recently demonstrated in a study exploring CD28-blocking antibodies (44). Lesional T cells were found to be dependent on CD28-mediated co-stimulation, even more so than normal control T cells. Blocking the CD28-CD86 receptor ligand interaction had profound inhibitory effects on the vasculitic process (44). Not only was co-stimulation relevant in determining the strengths of T cell activation, it regulated the amount of pro-inflammatory effector cytokines produced in the vasculitic lesions. Most importantly, inflammation-induced remodeling of the vessel wall, involving intimal hyperplasia and neoangiogenesis, required crosslinking of CD28 by CD80/CD86 (44). Taken together, by controlling *in situ* co-stimulatory signals, vasDC ultimately shape several dimensions of the vasculitic process (**Figures 2** and **4**).

The dynamics and intensities of T cell activation not only depend on co-stimulation but are equally shaped by co-inhibitory signals. In healthy arteries, vasDC express the inhibitory ligand PD-L1, effectively dampening T cell triggering. It is now recognized that a defect in this co-inhibitory pathway is a hallmark of GCA (45). Specifically, DC-Sign^+^ vasDC in healthy temporal arteries not only express CD80/CD86, but also PD-L1 (**Figures 2** and **5**). Crosslinking of the PD-1 receptor on T cells may indeed be one of the mechanisms through which vasDC protect the tissue niche and interrupt *in situ* immune activation. In GCA, vasDC lack PD-L1, suspending a negative feedback mechanism that halts inappropriate T cell stimulation. The PD-L1^lo^ phenotype is shared amongst patient-derived DC and macrophages (30), indicating a fundamental breakdown of this important immune checkpoint. The lack of PD-L1 in inflamed arteries does not explain why essentially all CD4^+^ T cells in the lesions are strongly positive for PD-1 (45). Possible explanations are that under physiologic conditions negative signaling induced by tissue-expressed PD-L1 prevents access of T cells to the tissue niche and that this mechanism is defective in PD-L1^lo^ hosts.

The PD-1/PD-L1 immune checkpoint is a critical regulator of immunity and is now one of the most important therapeutic targets in cancer patients (46). PD-1/PD-L1 deficiency in GCA has two clinically relevant consequences (47). First, excessive activity of this checkpoint is linked to insufficient anti-tumor immune responses. Tumors aberrantly express PD-L1 and utilize this mechanism to escape from anti-tumor T cell immunity. The defect of PD-1/PD-L1 signaling in GCA and the excess of PD-1/PD-L1 signaling in cancer patients raises the question whether GCA patients have a natural protection from malignancy. Epidemiological studies support the concept that GCA patients die from cancer less than expected (48, 49). If the broken PD-1/PD-L1 checkpoint has beneficial effects for GCA patients, then therapeutic efforts to restore the checkpoint could be effective to inhibit vasculitis while enhancing the risk for malignancy. This is not a trivial consideration, given the advanced age and the age-related cancer risk in patients with GCA. Vice versa, weakening the PD-1/PD-L1 checkpoint is the therapeutic goal in the widespread application of checkpoint inhibitors in patients with malignancies. This therapeutic intervention should place the host at risk to develop vasculitis. In support of this concept, numerous case reports have described aggressive aortitis and vasculitis in checkpoint inhibitor treated individuals (50, 51). In a human artery-SCID mouse chimeric system, in which human arteries are engrafted into NSG mice and vasculitis is induced by adoptive transfer of peripheral blood mononuclear cells of GCA patients, injection of a PD-1 blocking antibody produced aggressive vessel wall inflammation and vascular remodeling (45). More importantly, healthy mononuclear cells were able to induce vasculitis, if the checkpoint was blocked. This mimics conditions in checkpoint inhibitor treated cancer patients and emphasizes the risk of such cancer patients to come down with iatrogenic vasculitis.

Recent data suggest that a second immunoinhibitory checkpoint involving V-domain Immuno-globulin-containing suppressor of T cell activation (VISTA) may be less functional in GCA (52). Hid Cadina et al. have reported that VISTA+ Th cells are reduced in the blood of GCA patients but enriched in the inflamed temporal arteries.

Taken together, DC and other antigen-presenting cells make critical contributions to GCA, not only by *in situ* antigen presentation, but by distorting the threshold settings for T cell activation (**Figures 2**, **4** and **5**). GCA DC drive vasculitis by expressing CD86, amplifying disease-relevant enhancement of T cell immunity. At the same time, they fail to dampen lesional T cells by lacking PD-L1 on their surface. PD-L1^lo^ DC allow tissue entrance and persistence of highly activated effector T cells. The disbalance between robust co-stimulation and ineffective co-inhibition sets the stage for uncontrolled T cell immunity, with all the sequelae of a maladaptive response-to-injury. This scenario also presents an untapped therapeutic opportunity: treating GCA by interrupting excess co-stimulation or by reinstating co-inhibition (**Figure 5**). To which degree DC participate in the extravascular disease pathways of GCA is currently unknown. It is possible that DC in the circulation or in non-vascular tissues also have disease relevance and that they collude with abnormal T cells to render individuals susceptible to vasculitis (40).

### Other Innate Cell Types

Other types of innate immune cells, such as neutrophils, eosinophils and NK cells are typically scarce or absent in the vasculitic lesions of GCA (53). Indeed, eosinophilic inflammation should prompt the search for an alternative diagnosis, such as eosinophilic granulomatosis with polyangiitis. In rare cases, temporal arteritis can be attributed to an alternative vasculitis and atypical features on histologic examination are often the first clue (54, 55). Neutrophils may have a role in extravascular GCA. In the peripheral blood of GCA patients, decrease of suppressor neutrophils has been reported to accelerate effector T cell proliferation (56). GCA shares with other vasculitides the presence of immature neutrophils in the peripheral blood, which tightly correlated with inflammatory activity. In an *in vitro* co-culture system, such immature neutrophils produced abundant reactive oxygen species that caused protein damage and injured the endothelial barrier permeability (57). Mast cells may play an active role in vessel wall inflammation and have been described as one of the cellular sources of VEGF in temporal arteritis lesions (58).

## Adaptive Immunity in GCA

GCA is an HLA class II associated disease and the dominant cell type in the vasculitic lesions are CD4^+^ T cells, moving the adaptive immune system into center stage (**Figures 1** and **3**). Immunophenotyping of inflamed arteries demonstrates that CD4^+^ T cells outnumber CD8^+^ T cells (**Figures 1** and **3**), a feature which distinguishes GCA and Takayasu arteritis (9). In line with the observation that HLA class I molecules seem to be important as disease risk markers in Takayasu arteritis, the cytotoxic functions of CD8^+^ T cells have been implicated as a relevant disease mechanism in this vasculitis (9). However, recent data have assigned a disease relevant role to CD8^+^ T cells in the periphery. Specifically, CD8^+^ T cells with regulatory function, CD8^+^ Treg cells, are defective in GCA patients, failing to dampen CD4^+^ T cell function in vasculitis (59, 60).

Granulomatous infiltrates are typically composed of CD4^+^ T cells and macrophages and contain few B cells (61). Accordingly, autoantibodies seem to play no role in GCA. Cellular accumulations reminiscent of tertiary lymphoid aggregates, including B cells and plasma cells, have been seen in GCA affected aortic tissues (62). Whether they have functional relevance remains to be determined, but B cells are not recognized as drivers of the typical granulomatous reaction causing GCA. It is to be expected that systemic inflammation and the acute phase reaction typical for extravascular GCA leads to shifts in the distribution of circulating B cells (63). However, the biological relevance is unknown.

Sharing of T cell receptor sequences in independent tissues sites affected by GCA has nurtured the concept that antigen recognition is central in the emergence of the granulomatous lesions (64, 65). The nature of a causative antigen, however, has remained speculative. A tempting speculation is the proposal to implicate viral infections. Elderly individuals harbor a spectrum of chronic viral infections and the immune aging process makes them more susceptible to reactivation (66). Thus, it has been proposed that varicella zoster may be the underlying trigger of GCA, but carefully designed studies have refuted this theory (67–69). Recent observations that cancer patients treated with immune checkpoint inhibitors are at high risk to develop therapy-induced vasculitis (9, 50, 51) have emphasized the role of antigen-nonspecific mechanisms. If unleashed, polyclonal T cell populations appear to be able to promote vasculitis.

At the current stage of knowledge, T cells in GCA patients make several mistakes that culminate in loss of tolerance and the establishment of chronic-persistent inflammatory infiltrates in the wall of susceptible arteries.

### Peripheral CD4+ T Cells in GCA Patients

The hallmark abnormality in circulating CD4^+^ T cells from GCA patients is the aberrant expression of NOTCH1 (24, 70) (**Figure 2**). *NOTCH1* is an oncogene, most notably, *NOTCH1* mutations are present in the majority of patients with T cell acute lymphocytic leukemia (71). NOTCH signaling controls cell fate decisions and is needed for the specification of T cells; directs cell proliferation, differentiation, and cell death (72, 73). In GCA, NOTCH1 expression on circulating CD4^+^ T cells has been implicated in enabling their transition from the blood into the tissue, representing a major tolerance defect in this disease. CD4^+^NOTCH1^+^ T cells from GCA patients recognize aberrantly expressed JAGGED1 on the surface of vasa vasorum endothelial cells (24), facilitating their invasion into the vessel wall (**Figure 2**). Targeting the NOTCH1-JAGGED1 interaction was sufficient to suppress vasculitic activity (24), placing this receptor-ligand interaction at the top of GCA pathophysiology. A prerequisite for the transmigration from the capillary lumen into the perivascular space is the action of MMP-9-expressing monocytes, which first must digest the basal lamina to pave the way for T cells (14) (**Figure 2**). In the absence of monocytes or macrophages, GCA T cells fail to invade into 3D extracellular matrix. The dependence of T cells on pathogenic monocytes/macrophages exemplifies the co-occurrence of abnormalities in the innate and adaptive immune system steering inflammatory cells into an immunoprivileged tissue site.

### Tissue-Residing CD4^+^ T Cells in GCA Lesions

**Figure 6:**
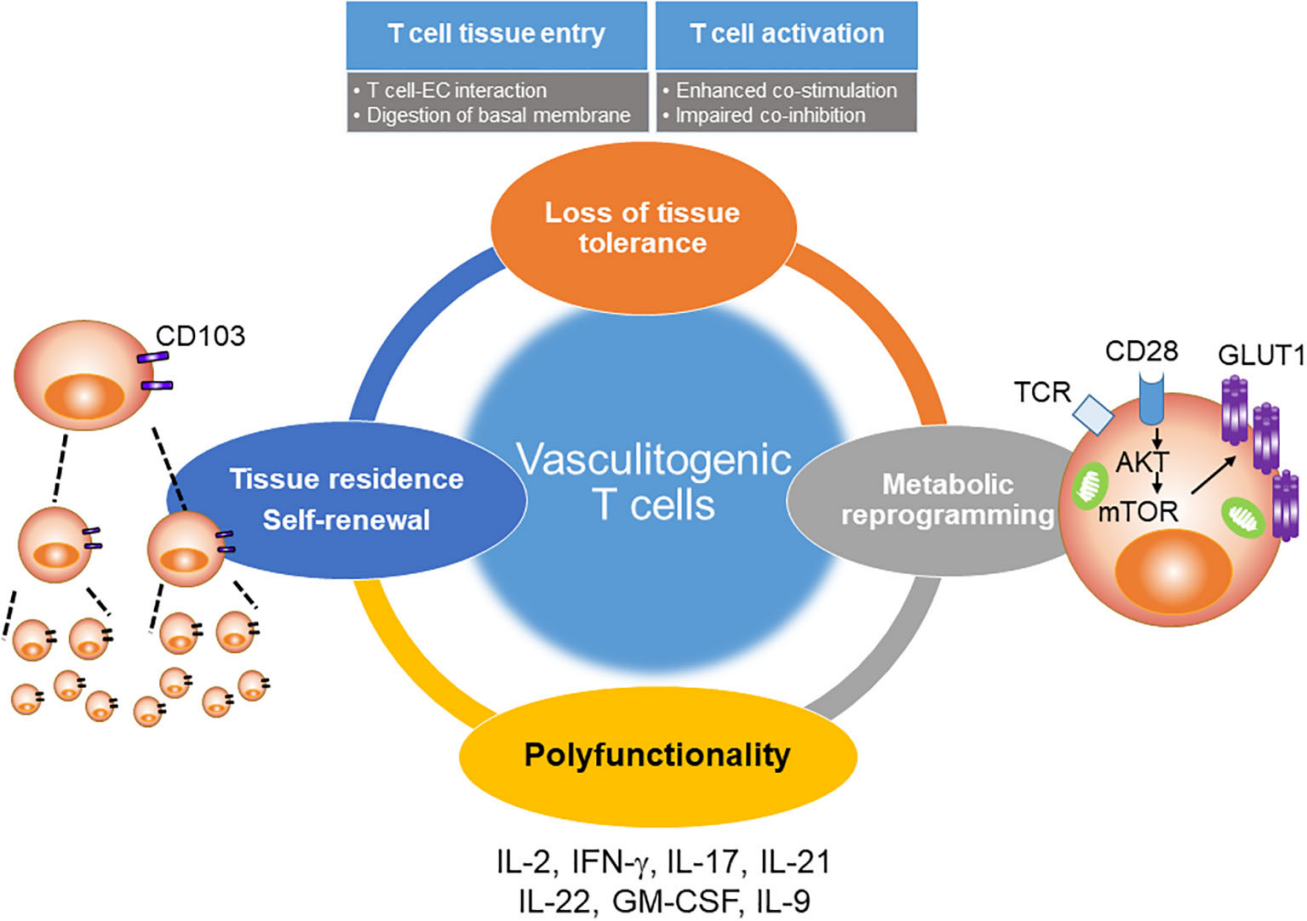
Pathogenic role of T cells in GCA. The shift from protective to pathogenic immunity in GCA involves multiple processes. Four major abnormalities have been molecularly defined (1). In GCA patients, CD4+ T cells aberrantly express NOTCH1, which facilitates T cell-endothelial communication, tissue entry and uncontrolled T cell expansion (2). Excess CD28-dependent signaling imprints a metabolic signature that sustains pro-inflammatory T cells (3). Such T cells supply a multitude of effector cytokine to stimulate macrophages and vascular cells (4). A critical feature of disease-promoting CD4^+^ T cells is the ability to establish tissue residency in the vessel wall. Tissue-resident memory CD4^+^ T cells render the lesion autonomous and ensure chronicity of disease.

Lesional CD4^+^ T cells in the vasculitic wall have two major abnormalities; they are pluripotent effector cells, supporting a multitude of inflammatory effector pathways and they can self-renew to sustain the wall infiltrates and turn acute vasculitis into chronic-persistent disease (**Figures 5** and **6**).

The vast majority of lesion-residing CD4^+^ T cells are strongly positive for PD-1 (45) (**Figure 5**). On human T cells, PD-1 is an activation marker, but more importantly, has been implicated in tumor evasion mechanisms and in exhaustion of chronically stimulated T cells (74). CD4^+^ T cells trapped in the arterial wall are not exhausted, nor are they senescent. Rather, their accumulation is a consequence of insufficiency in PD-L1 expression (see above). Both, vascular DC, and macrophages are distinctly low for PD-L1, disrupting a negative signal to PD-1^+^ T cells. The entry of T cells into the wall and the accumulation/retention of T cells in the wall are both dependent on PD-L1 (45).

Besides expressing surface PD-1, lesional CD4^+^ T cells are polyfunctional (45) (**Figure 6**). A multitude of T cell effector cytokines have been mapped to the lesions, including IL-2, IFN-γ, IL-17, IL-21, IL-9, IL-22, and GM-CSF (**Figure 6**). It has not been clarified whether the polyfunctionality occurs on the level of individual cells or the T cell population. IFN-γ-producing CD4^+^ T cells represent the dominant T cell subset in inflamed temporal arteries (11, 75). IFN-γ-producing CD4^+^ T cells are expanded in the peripheral blood of GCA patients and are resistant to corticosteroid therapy (75). IFN-γ has all the characteristics of a critical effector cytokine, as it activates macrophages, DC, and endothelial cells. Interestingly, IFN-γ^+^ CD4^+^ T cells map preferentially to the adventitia of GCA-affected temporal arteries (76). How they guide the activity of the granulomatous infiltrates needs to be clarified. However, the geographical distance to migrating myofibroblasts may be important, as IFN-γ is considered to inhibit proliferation of mesenchymal cells.

In contrast to their resistance to corticosteroids, IFN-γ^+^ T cells are dependent on Janus kinase (JAK) and signal transducer and activator of transcription (STAT) signaling and this dependence creates a vulnerability that can be therapeutically exploited. Inhibiting the JAK/STAT signaling pathway with a small molecule inhibitor targeting JAK1/3 is highly effective in suppressing vasculitis, including the IFN-γ-producing CD4^+^ T cells (77). These data have raised the possibility that IFN-γ production is part of a feed forward loop, as IFN type II is a potent inducer of JAK/STAT signaling (78). Tissue transcriptomic studies have indicated that STAT target genes are strongly upregulated in the lesions, including target genes of IFN type 1 and type 2.

IFN-γ-producing CD4^+^ T cells are accompanied by subsets of lesional T cells that produce IL-17, IL-21, and IL-9 (79, 80). Likely, each of these T cell lineages makes a specialized contribution to the disease process, but mechanistic studies detailing this are not yet available. IL-17^+^ T cells in GCA lesions have been reported to be highly sensitive to corticosteroid therapy, disappearing upon initiation of this immunosuppressant (75) and are thus different from IFN-γ^+^ T cells, that persist over prolonged periods despite steroid therapy (75). Th17 cells may thus be easily controllable and may not have much value as a therapeutic target.

IL-21-producing CD4^+^ T cells are abundant in the tissue lesions and in the blood of patients with GCA and appears to be sensitive to glucocorticoid treatment (79). IL-21 is reported to play a role in supporting Th1 and Th17 responses and suppressing FOXP3^+^ T regulatory cells in GCA (79), but the precise pathogenic role of IL-21 remains unclear.

IL-9 is a pleiotropic cytokine, with the potential to drive both pro-inflammatory and anti-inflammatory responses (81). High expression of IL-9 was reported in temporal artery biopsies (80), but how this cytokine influences vasculitic immune responses is unknown.

IL-22 is believed to mediate the crosstalk between immune cells and stromal cells (82). IL-22 has been encountered in temporal artery biopsies and is strongly linked to vasculitis (83). Little is known so far how stromal cells are involved in the disease process, but they are ultimately important in wall remodeling. Whether IL-22-dependent immunity is relevant in the maladaptive wound healing response awaits clarification.

The T cell effector cytokine GM-CSF is considered an important regulator of macrophages (84) and could provide effective T cell-macrophage communication in the granulomatous infiltrates (84). Indeed, macrophages activated by GM-CSF acquire numerous effector functions, enabling them to amplify tissue inflammation. GM-CSF is the product of a specialized T cell subset that has high disease relevance in multiple sclerosis (85, 86).

### Tissue-Resident Memory T Cells in the Inflamed Artery

Despite the physiological ability of host immune protection by trafficking of memory T cells around the body, recent studies have revealed that specialization of pathogenic memory T cells into unique tissue-resident subsets may drive regional autoimmunity (87, 88). Long-lasting immunity causing temporal artery damage is mediated by tissue resident memory T cells (44, 77). Data from re-engraftment studies have revealed that vasculitis-causing T cells acquire tissue residency and build autonomous, self-sufficient inflammatory lesions (77), where repopulation of inflammatory CD4^+^ T cells is maintained from tissue-resident memory populations (**Figure 6**). Further, metabolic analysis of tissue resident memory T cells in the vasculitic wall lesions has yielded evidence for high glycolytic activity resulting from CD28-dependent signals and fulfilling the energy demand of repopulating effector T cells (44). Those tissue-residing T cells are polyfunctional and steroid therapy resistant. In fact, a study analyzing temporal artery biopsies before and up to 12 months after steroid therapy found that half of GCA patients still have ongoing vessel wall inflammation after one year of immunosuppression (13).

### CD8^+^ T Cells in GCA

Early studies examining frequencies of circulating CD8^+^ T cells in GCA gave rise to the hypothesis that a reduction of CD8^+^ T cells is typical for active untreated GCA (89). This hypothesis was called into question by later studies (90). A recent manuscript described altered gene expression profiles in blood CD4 and in CD8 T cells in a cohort of 16 GCA patients that were monitored by longitudinal expression profiling (91).

A clue towards an entirely new disease mechanism in GCA CD8 T cells has come from studying the T cell aging process. T cell aging leads to a maladaptive response that directly contributes to chronic inflammatory disease (92). CD8^+^ T cells are well-known to age faster than CD4^+^ T cells and a hallmark of T cell aging is the loss of naïve CD8^+^ T cells (93). In fact, older individuals fail to generate CD8^+^ CCR7^+^ T regulatory cells, rendering them susceptible to unopposed immune reactivity (59). Age-dependent decline of protective immunity and rise of dysfunctional immunity may be one of the reasons that GCA occurs exclusively in individuals older than 50 years of age. Indeed, loss-of-function of protective CD8^+^ Treg cells is associated with aging (59). Mechanistically, CD8^+^ T regulatory cells suppress activation and expansion of CD4^+^ T cells by releasing exosomes that contain preassembled NOX2 membrane clusters which are taken up by CD4^+^ T cells (59). Defective CD8^+^ T regulatory cells in GCA patients lose the ability to package NADPH oxidase into immunosuppressive exosomes. A recent study has identified the molecular mechanism leading to CD8^+^ Treg cell failure in GCA patients (60). The inability of GCA CD8^+^ Treg cells to release NOX2-containg, immunosuppressive exosomes was mechanistically connected to abnormalities in endosomal trafficking. Specifically, due to aberrant NOTCH4 signaling, GCA CD8^+^ Treg cells changed the profile of RAB GTPases, which promoted NOX2 trapping in an intracellular compartment of early and recycling endosomes (60).

These studies have identified a novel molecular abnormality linking T cell aging, Treg cell failure and susceptibility to vasculitis. Implicating RAB GTPases and intracellular vesicular trafficking in disease pathogenesis opens new conceptual and therapeutic opportunities.

## From Bench to Bedside: Potential Therapeutic Targets in GCA

Glucocorticoids (GC) remain the standard therapy, possibly because of their untargeted immunosuppression and the multiplicity of pathogenic pathways contributing to GCA (**Figures 2**, **5**, **6**). GC are highly effective in suppressing extravascular GCA, flattening the acute phase response, clinical symptoms, and abnormal laboratory parameters (94). To examine the remission-inducing potential for the vessel wall component of the disease, we have utilized a dual-biopsy approach. 40 patients with a positive temporal artery biopsy received standard doses of prednisone and were re-biopsied on the collateral side at 3, 6, 9 or 12 months (13). About 50% of patients had active vasculitis after 12 months of GC therapy (13). Patients with a positive second biopsy had excellent clinical and laboratory responses and were clinically indistinguishable from patients in whom the second biopsy was negative. Thus, GC therapy is highly efficient for extravascular GCA and insufficiently treats vascular GCA. Also, clinical assessment and monitoring of sedimentation rate and CRP are not able to assess the inflammatory load in the vessel wall. Overall, new therapeutic approaches are needed to treat GCA, probably in form of combination therapy. The resistance of the vascular component, likely a consequence of the ability of the disease lesions to become autonomous, emphasizes the need for more efficient immunosuppression that can be given over extended time periods in elderly individuals. New therapeutic strategies need to go hand-in-hand with the development of diagnostic tools that allow quantification of vessel wall inflammation.

Tocilizumab, an anti-IL-6 receptor antibody, has shown efficacy in suppressing ESR and CRP, helping to spare GC dosing to manage the acute phase response (95). However, it remains unknown whether inhibiting IL-6 signaling has beneficial effects on vessel wall inflammation itself. In fact, discrepancies between vascular and extravascular inflammation in large vessel vasculitis has been increasingly recognized and represents the most challenging problem in the management of this autoimmune vasculopathy (96). Disease flares are frequently observed even in GCA patients treated with tocilizumab plus GC that have reached normal acute-phase reactant levels. Further, disease progression of local vessel wall inflammation has been reported in patients with Takayasu arteritis on tocilizumab treatment although they were clinically asymptomatic and had normal laboratory findings (97–99). Patients with Kawasaki disease on tocilizumab treatment have been reported to develop giant coronary aneurysms despite clinical and laboratory improvements (100). These data are in line with the concept that correcting downstream inflammatory parameters is insufficient to reset upstream abnormalities in the immune system of the patients. Here, progress made in understanding the immune signaling networks underlying vascular inflammation needs to guide the exploration of novel therapeutic interventions, including those intended to control the inflammatory attack of the vessel wall. One possible approach is to target key effector cells in the vascular lesions, e.g. macrophages. Currently ongoing trials with the GM-CSF receptor blocker mavrilimumab are designed to disrupt the inappropriate macrophage activation in the lesions. An alternative approach is to interfere with disease relevant signaling pathways, which may be shared by several cell populations relevant in the disease process.

Here, we have summarized the signaling pathways that are now understood to contribute to the immunopathogenesis of GCA and may serve as therapeutic targets (**Table 2**).

**Table 2:** Potential therapeutic targets in giant cell arteritis.

Targets	Pathogenic role in vessel wall inflammation	Drugs
mTOR signaling	T cell proliferation and survival; Metabolic control of T cell effector differentiation and of T cell functions;	Rapamycin
VEGF signaling	Endothelial cell homeostasis; Maintenance of vasa vasora; Pathogenic wall vascularization; Induction of co-stimulatory ligands (Jagged1);	Bevacizumab
NOTCH signaling	T cell fate decisions; T cell co-stimulation; T cell clonal expansion and survival; T cell tissue invasion; Trafficking of intracellular vesicles;	DAPT
JAK-STAT signaling	Type I and type II IFN-dependent responses;	Tofacitinib Baricitinib
CD28-AKT signaling	Uncontrolled co-stimulation; Metabolic programming of effector T cells;	Abatacept Anti-CD28
PD-1/PD-L1 signaling	Deficient co-inhibition; Failure of negative signaling; Inappropriate T cell expansion, survival and effector functions;	PD-L1 Fc PD1 agonists
MMP-9 production	Destruction of the arterial wall tissue barrier; Structural damage to the vessel wall;	MMP-9 blockade

### mTOR Signaling

The serine/threonine kinase mTOR (mechanistic target of rapamycin) is designed to integrate environmental signals to coordinate cellular response patterns. mTOR is a critical signaling hub in all cell types relevant for vasculitis, including T cells, which rely on mTOR activity for their development, differentiation functional fitness. mTOR signaling guides effector cell fate decisions, a fundamental abnormality in T cells from GCA patients. mTOR has also been implicated in controlling the suppressive activity of regulatory T cells and regulates the process of T cell exhaustion. Aberrant mTOR activation is a hallmark abnormality in CD4^+^ T cells from GCA patients, both in circulating as well as lesional T cells (24, 101). mTOR signaling may also be a driving force in endothelial cells of microvessels that provide access to the vessel wall and function as partners of effector T cells (101). mTOR functions as a sensor of nutrient resources, particularly amino acid supply (102). GCA T cells utilize a highly activated glycolytic program to support their effector functions and sustain their self-replicative potential (44). Inhibiting mTOR signaling may therefore disrupt an array of disease-relevant T cell functions. The wall remodeling process is dependent on cellular growth and expansion, with multiple cell types involved. mTOR activity may represent a common denominator driving cellular activity of diverse pathogenic population and as such represent a unifying target to treat vasculitis.

### VEGF-NOTCH Signaling

Serum VEGF levels are highly elevated in patients with GCA, indicating the critical role of angiogenesis and endothelial cell function in this autoimmune vasculitis (21, 24). Endothelial cells lining the vasa vasorum are the gate keeper of the vessel wall and this barrier has been overcome to enter the tissue niche (8). Also, the remodeling process is particurly dependent on neoangiogenesis within the wall layers. Macrophages, multinucleated giant cells, and mast cells have been identified as a cellular source of VEGF in GCA. VEGF functions not only as an angiogenic factor but also activates endothelial cells, upregulating Jagged1 expression on adventitial vasa vasorum endothelial cells, thus turning the EC into an engaged partner to interact with NOTCH 1 receptor-expressing T cells (24). The surplus of VEGF promotes endothelial cell proliferation and sustains formation of new capillaries (103, 104). Anti-VEGF treatment is widely applied in the therapy of malignant tumors and has become a promising treatment to inhibit aberrant neovascularization in ocular disease (105). The NOTCH signaling pathway has been investigated as a therapeutic target to block proliferative activity in malignant cells (106). Here, GCA displays abnormalities that are shared between cancer and autoimmunity, encouraging the exploration of anti-angiogenic and anti-proliferative interventions in GCA. Stopping angiogenesis may have benefit in dampening wall remodeling. Inhibiting NOTCH signaling may prevent aberrant cellular activation for multiple disease relevant cell populations.

### JAK-STAT Signaling

Transcriptomic analysis has shed light on ongoing JAK-STAT signaling in inflamed temporal arteries, implicating mostly Type I and Type II IFN-dependent responses (77). Type II IFN-regulated inflammation is in line with the critical position of IFN-γ in disease pathogenesis. Little is known about a potential role of IFN type I. Notably, upregulation of Type I and Type II IFN as upstream inducers of pathogenic immunity has also been reported for Takayasu arteritis (107). We have published a proof-of-principle study showing that treatment with tofacitinib, a selective JAK1 and JAK3 inhibitor, is highly efficient in suppressing vessel wall inflammation (77). Unexpectedly, interfering with JAK-STAT signaling was highly successful in interrupting both intimal hyperplasia and wall capillarization in the human artery -SCID chimera model (77). Recently reports suggest that tofacitinib may have a place in managing patients with refractory Takayasu arteritis (108). These data support the concept that autoimmunity in the wall of large arteries relies disproportionally on JAK-STAT signaling and that therapeutic opportunities lie in dampening excessive activity in these fundamental signaling pathways.

### T-Cell Co-stimulation and Co-inhibition

GCA is a granulomatous vasculitis, defining T cells and macrophages as the key pathogenic drivers. The intensity and duration of T cell activation is not only dependent on antigen recognition, but equally important are co-stimulatory and co-inhibitory signals. Recent data support a role for CD28-mediated co-stimulation in several domains of the disease process, including T cell expansion, survival, and metabolic fitness. Randomized controlled trials have reported the efficacy and safety of abatacept, CTLA-4Ig, that blocks T cell co-stimulation in patients with GCA (109). However, these clinical trials have focused on assessing inflammatory markers (ESR, CRP) and clinical relapses, which all reflect activity in the extravascular arm of GCA. Data are needed to understand whether blocking CD28 co-stimulation has beneficial effects for chronicity of vascular inflammation and the adverse remodeling process of the vascular wall. Proof-of-principle studies in humanized mice are encouraging, emphasizing the dependence of wall inflammation on CD28-mediated co-stimulatory input (44).

Under physiologic conditions, co-stimulatory signals are offset by co-inhibitory signaling. Amongst the inhibitory pathways, the PD-1/PD-L1 pathway is best known due to the aberrant expression of PD-L1 on tumor cells, which paralyses anti-tumor T cell responses. The PD-1/PD-L1 pathway is deficient in GCA due to the low PD-L1 expression on the patients’ dendritic cells and macrophages (45). Numerous therapeutic antibodies are in use to disrupt PD-1/PD-L1 signaling in cancer patients, but so far, no therapeutics are available to strengthen PD-1 signaling. Options include agonistic anti-PD-1 antibodies, transferring negative signals into T cells or replacing the lacking PD-L1 with soluble PD-L1 fused to an Fc domain.

### Excess Production of the Metalloproteinase MMP-9

Breakdown of the basal lamina, enabling the transition of macrophages and T cells out of the blood stream into the extracellular space of the vessel wall, is an early pathogenic event in GCA and depends on MMP-9-mediating digestion of the protective basal membrane (14). In a preclinical model system, treatment with an antibody blocking MMP9 activity was sufficient to halt vasculitis and prevent vessel wall remodeling (14). An appealing aspect of targeting MMP-9 lies in the potential to stop invasion of the vessel wall while protecting the immunocompetence of the host. MMP-9 was detected in three cell populations: monocytes, macrophages, and multinucleated giant cells. Interfering with the activity of MMP-9 would thus provide opportunities to target innate immunity in GCA, while preserving adaptive immunity. Also, MMP-9 participates in very early steps of autoimmune vasculitis and may be able to terminate invasion of the artery. At the same time, MMP-9 is a key molecule in the destruction of elastic membranes and may be particurly important in complication of GCA aortitis, such as wall dissection and aneurysm formation (14). Finally, MMP-9 blocking agents may be best placed in combination therapies that use a two-pronged approach to have an impact on the complex pathogenesis of GCA.

### IL-12/IL-23 Signaling

A hallmark of GCA is the recruitment and retention of highly differentiated effector T cells that become part of the granulomas (**Figure 5**). The differentiation process depends upon lineage-inducing cytokines, such as IL-12 and IL-23, which are major regulators of T cell fate. IL-12 and IL-23 have been implicated in promoting Th1 and Th17 lineage commitment in both GCA and Takayasu arteritis (75, 110). In situ IL-12 and IL-23 heterodimers have been reported in temporal arteritis lesions (111). In addition, genome-wide association studies have categorized IL-12B as a susceptibility gene for Takayasu arteritis (112, 113). Ustekinumab, a monoclonal antibody that inhibits both IL-12 and IL-23 signaling by binding to the common p40 subunit, has been tested in patients with GCA and Takayasu arteritis (114, 115). A prospective, open-label trial of ustekinumab in 13 patients with active new-onset or relapsing GCA was prematurely closed because patients could not reach prednisone-free remission (116). Blocking IL-12/IL-23 should interfere with the differentiation program of naïve into memory/effector T cells, a process that may precede the onset of vasculitis. Given the autonomy of the vascular lesions (see above tissue-resident memory T cells), interfering with the IL-12/IL-23 pathway may need to be combined with blocking the primary seeding of the vessel wall.

## Conclusions

Autoimmune disease infrequently targets arteries, but autoimmune vasculitis is a dangerous disease due to the high potential for life-threatening complications. Large arteries, such as the aorta, respond to autoimmune attack with loss of wall integrity, clinically presenting as dissection, aneurysm formation of rupture (**Figure 3**). In medium arteries, wall inflammation results in a maladaptive remodeling process that occludes the lumen and causes tissue ischemia (**Figure 1**). The pathologic lesion is a granulomatous reaction, often with formation of multinucleated giant cells (**Figures 1** and **3**). The molecular signature of disease-relevant monocytes and macrophages includes the aberrant production of the metalloproteinase MMP-9 and the selective loss-of-function of the inhibitory ligand PD-L1. In the vasculitic lesions, macrophages are critical effector cells, supplying cytokines, metalloproteinases and angiogenic factors. The therapeutic targeting of pathogenic macrophage functions is only superficially explored but holds promise to provide entirely new strategies for anti-vasculitic immunotherapy (**Figure 2**).

As documented by the granulomatous nature of autoimmune vasculitis, GCA is ultimately a disease of misdirected adaptive immunity. The master regulators of the faulty immune response are CD4^+^ T cells that enter a protected tissue niche, take tissue residence, gain autonomy, and differentiate into multiple classes of differentiated effector T cells (**Figure 6**). Accordingly, the vasculitic lesions are rich in a spectrum of effector cytokines, including IL-2, IL-9, IL-17, IL-22, GM-CSF, IFN-γ, and IL-21. Each of these effector cytokines contributes in its own right, multiplying the pathogenic potential of T cell accumulations forming within the layers of the arterial wall. The multiplicity of effector T cell populations makes a single causative antigen highly unlikely.

The molecular signature of pathogenic CD4^+^ T cells in GCA includes the aberrant expression of the NOTCH1 receptor, and the reliance on CD28 costimulatory signaling unopposed by PD-1 inhibitory signaling (**Figures 2** and **6**). GCA patients have metabolically active CD4^+^ T cells with persistent mTORC1 activation. These T cells are powerful drivers of pathogenic cascades that finally lead to wall destruction or to intimal hyperplasia and luminal occlusion. The complexity of GCA pathogenesis offers multiple intersection points that should allow to broaden the diagnostic and therapeutic approach to this difficult-to-manage autoimmune disease (**Figure 6**; **Table2**). A major hurdle lies in the split of the disease process into an extravascular and a vascular component which are at least to a large extent independent of each other. Extravascular and vascular GCA follow different trajectories, relate to different pathogenic mechanisms and ultimately, require different diagnostic and therapeutic schemes.

Several unanswered questions remain. How does the aging process of the vessel wall and the immune system conjoin to render the host susceptible to GCA? How does the tissue microenvironment create the stringent tissue tropism of this autoimmune disease? Are there vasculitogenic antigens or is the fundamental abnormality solely a defect in threshold setting of CD4^+^ T cells? How do CD4^+^ T cells engage vascular stromal cells to cause intimal hyperplasia? What is the underlying mechanism driving T cell polyfunctionality? What are the pathogenic processes underlying extravascular GCA? A new conceptual approach to this autoimmune and autoinflammatory condition will pave the way to the development of novel diagnostic and therapeutic modalities.

## Author Contributions

MA, JG, and CW wrote the manuscript. SO and GB contributed figures. The concept presented in the manuscript were developed by CW, JG, GB, and DL. All authors contributed to the article and approved the submitted version.

## Funding

This work was supported by the National Institutes of Health (R01 AR042527, R01 HL117913, R01 AI108906, R01 HL142068, and P01 HL129941 to CW and R01 AI108891, R01 AG045779, U19 AI057266, and R01 AI129191 to JG) and Merit Review Award I01 BX001669 from the United States (U.S.) Department of Veterans Affairs to JG. The contents do not represent the views of VA or the United States Government. MA was supported by a research fellowship from the Uehara Memorial Foundation.

## Conflict of Interest

CW was supported by a sponsored research agreement with Kiniksa Pharmaceuticals.

The remaining authors declare that the research was conducted in the absence of any commercial or financial relationships that could be construed as a potential conflict of interest.
